# *SAMHD1* Mutations and Expression in Mantle Cell Lymphoma Patients

**DOI:** 10.3389/fonc.2021.763151

**Published:** 2021-12-17

**Authors:** Tao Wang, Wenqin Yue, Gusheng Tang, Mingyu Ye, Jiechen Yu, Bin Liu, Lijuan Jiao, Xuefei Liu, Shuyi Yin, Jie Chen, Lei Gao, Jianmin Yang, Miaoxia He

**Affiliations:** ^1^ Department of Hematology, Changhai Hospital, Second Military Medical University, Shanghai, China; ^2^ Department of Pathology, Changhai Hospital, Second Military Medical University, Shanghai, China

**Keywords:** SAMHD1, mantle cell lymphoma, cytarabine resistance, immunohistochemistry, mutations, prognosis, gene silencing, patient risk stratification

## Abstract

SAMHD1 (sterile alpha motif domain and histidine-aspartate domain-containing protein 1) is a deoxynucleoside triphosphate triphosphohydrolase regulating innate immune and modulating DNA damage signaling. It plays an important role in the development of some tumors. SAMHD1 was also reported as a barrier to cytarabine, a common chemotherapy drug for mantle cell lymphoma (MCL), and as a biomarker of grim prognosis for acute myelocytic leukemia (AML) patients. However, SAMHD1 expression and function in MCL have not been well-defined. In the present study, we evaluated SAMHD1 expression by immunohistochemistry and its gene structure by Sanger sequencing in MCL. Our results showed that SAMHD1 was positive in 36 (62.1%) patients. Importantly, SAMHD1-positive patients were associated with lower chemotherapy response rate (*p* = 0.023) and shorter overall survival (*p* = 0.039) than SAMHD1-negative cases. These results suggest that SAMHD1 is an adverse biomarker for MCL patients, which is due to the high expression of SAMHD1 and rapid cell proliferation. These findings were confirmed in an *in vitro* study using the *si*RNA technique. Silencing the *SAMHD1* gene in the MCL cell line Jeko-1 significantly decreased cell proliferation and increased cell apoptosis. The MCL cell line with *SAMHD1* knockdown showed lower Ki-67 proliferation index, higher caspase-3, and higher sensitivity to cytarabine. Furthermore, for the first time, four previously unreported missense mutations (S302Y, Y432C, E449G, and R451H) in exon 8 and exon 12 of the *SAMHD1* gene were discovered by sequencing. The mutations had not been found to corelate with SAMHD1 protein expression detected by immunohistochemistry. The biological functions of this mutated SAMHD1 remain to be investigated.

## Introduction

SAMHD1 (SAM And HD Domain-Containing protein 1) is a deoxynucleoside triphosphate (dNTP) triphosphohydrolase initially discovered in Aicardi-Goutie’s syndrome, a genetic encephalopathy (1). In 2011, SAMHD1 was identified as a restriction factor of human immunodeficiency virus-1 (HIV-1) replication in dendritic cells and myeloid cells for its inhibition of viral life circle by hydrolyzing dNTP into deoxynucleoside and inorganic triphosphate (2–4). Subsequently, SAMHD1 was reported to be responsible for maintaining cellular dNTP balance during the cell cycle and regulating the cell proliferation (5) as well as for DNA homologous recombination repair by promoting DNA end resection (6). Previous studies have identified aberrant SAMHD1 expression as a potential cause of colon cancer (7), lung cancer (8), chronic lymphocytic (B-cell) leukemia, and some subtypes of T-cell lymphoma or leukemia (9–11). Furthermore, *SAMHD1* mutations could be a founder event in chronic lymphocytic (B-cell) leukemia, and its potential role in cancer pathogenesis has been investigated. Most recently, SAMHD1 was reported as a barrier to cytarabine and a biomarker of grim prognosis for acute myelocytic leukemia (AML) patients (12). SAMHD1 could hydrolyze Ara-CTP in leukemic cells, resulting in the reduction of cellular Ara-CTP concentration and the attenuation of its efficacy (13, 14).

Mantle cell lymphoma (MCL) is a common subgroup of non-Hodgkin lymphoma (NHL) with distinctive clinical, biological, and molecular characteristics. The clinical management and prognosis of MCL are based on risk stratification, including the MCL International Prognostic Index (MIPI) and the Ki-67 proliferative index (15, 16). Besides the first-line chemotherapy regimens such as R-CHOP (rituximab, cyclophosphamide, doxorubicin, vincristine sulfate, and prednisone) and R-Hyper-CVAD (rituximab and hyper-cyclophosphamide, vincristine, adriamycin, and dexamethasone), it has demonstrated that chemotherapy with cytarabine could significantly improve the progression-free survival (PFS) and overall survival (OS) of the patient (17, 18). However, despite the standard chemotherapy with cytarabine, even combined with autologous stem cell transplant (19), the complete remission (CR) rate of MCL was still variable from 5% to 77% (18, 20–25). The 5-year PFS and OS were 11%–68% and 64%–77%, respectively (19, 26–32). Thus, further investigations are needed to identify therapeutic-related factors in MCL. Cytarabine was usually used to treat MCL in the standard chemotherapy regimen. However, the SAMHD1 status in MCL was rarely known.

In the present study, we assessed SAMHD1 expression and genetic mutations in MCL tissues. The association between SAMHD1 expression and chemotherapy response as well as clinical outcome was evaluated. Our findings provide novel insights of SAMHD1 influencing MCL for better clinical management.

## Patients and Methods

### Patient Selection

The patient database of the Department of Pathology, Changhai Hospital (Shanghai, China) was searched for MCL cases. Between January 2007 and January 2017, 58 patients were diagnosed as MCL in Shanghai Changhai Hospital. Pathological specimens were reviewed by two hematopathologists according to the 2017 updated World Health Organization (WHO) classification of tumors of hematopoietic and lymphoid tissue (33). Among these 58 patients, 33 patients received treatment in Changhai Hospital. Clinical data collected included age, gender, lactate dehydrogenase (LDH), beta2-microglobulin (β2-MG), hematological parameters, performance status, Ann Arbor stage, Eastern Cooperative Oncology Group (ECOG) performance status, MIPI, chemotherapy regimens, response to treatment, and outcomes.

This study was approved by the Institutional Review Board of Changhai Hospital. All patients provided written consent for the sample collection and use for research. The study was performed in accordance with the Declaration of Helsinki and was approved by the local ethics review committee.

### Immunohistochemistry

Tumor tissues were fixed using 10% neutral formalin followed by paraffin embedding. The formalin-fixed paraffin-embedded (FFPE) tissues were sliced into 3-µm-thick sections for routine H&E staining and two step Envision IHC. MCL diagnosis was based on morphology and immunophenotype. Antibodies used for the MCL diagnosis including those against CD20, BCL2, cyclin D1, SOX11, CD5, CD10, BCL6, MUM1, p53, and Ki-67. SAMHD1 expression in MCL was specially detected by immunohistochemistry (IHC). Anti-SAMHD1 (ab128107, Abcam, Cambridge, MA, USA) was used after 1:150 dilution. IHC results of cyclin D1, SOX11, CD5, CD10, CD20, BCL2, and BCL6 were recorded as negative or positive. SAMHD1 expression was scored as negative and positive (low ≤ 30%, moderate >30% to ≤60%, high > 60%), and Ki-67 was scored as low (0%–30%) and high (31%–100%).

### Fluorescence *In Situ* Hybridization

The translocation t(11;14)(q13;q32), cyclin D1 (CCND1)/immunoglobulin heavy chain (IGH), is a hallmark for MCL and was found in most of the cases (32). Recently, it was reported that some MCL of cyclin D1 negative had translocation of cyclin D2 or cyclin D3 with IGK or IGL partner (34, 35). Therefore, the fluorescence *in situ* hybridization (FISH) technique was used to detect the translocation/gene rearrangement of cyclin D1, cyclin D2, and cyclin D3. FFPE slides were deparaffinized with Hemo-De (Thermo Fisher Scientific, Waltham, MA, USA), rehydrated with gradient alcohols, pretreated with 10-fold diluted epitope retrieval solution (Dako North America Inc., Carpentaria, CA, USA) at 97°C for 25 min, digested with pepsin, and then hybridized respectively with dual color and dual fusion probe for t(11;14)(q13;q32) and break-apart probes for cyclin D2 and cyclin D3 (Abbott Molecular, Des Plaines, IL, USA) overnight. The next day, the slides were washed to remove excess FISH probes and counterstained with DAPI (Abbott Molecular, Des Plaines, IL, USA). FISH images were captured and analyzed by BioView Duret FISH analysis system (Billerica, MA, USA).

### Sanger Sequencing

The *SAMHD1* mutation was detected using Sanger sequencing. The reverse transcription technique was used to prepare cDNA from mRNA purified from MCL tumor tissues. Sixteen pairs of primers were designed to amplify the entire coding regions of *SAMHD1* (**Table 1**). Amplicons were purified and both strands were sequenced using Big Dye Terminator v3.1 kit (Thermo Fisher Scientific, Waltham, MA, USA) on ABI 3130 DNA sequencer (Thermo Fisher Scientific, USA). Sequencing results were analyzed by DNA variant analysis software (Thermo Fisher Scientific, Waltham, MA, USA).

**Table 1:** Primers used in *SAMHD1* sequencing.

Exon	Name	Primer sequence
1	Forward	5′-AGGTGCGGCGGGTAGTGTAC-3′
	Reverse	5′-CTTTCCTCGGCGCCCCCAGC-3′
2	Forward	5′-ATCCATTGCCTGCAGTGGGT-3′
	Reverse	5′-TCCAGCCTGGGTGAACAAGA-3′
3	Forward	5′-AGGACAGAAGGCTGTGGGAG-3′
	Reverse	5′-TGCAGAAAGTTTAGAAAAGATCCA-3′
4	Forward	5′-ATCAAATAGCTTTGACTTTGCACT-3′
	Reverse	5′-TCATGTGATCCACCCACCTC-3′
5	Forward	5′-TGCTTTTGGGATTCCGTTTG-3′
	Reverse	5′-GAATCATTCTAGGAAGAAGCAACA-3′
6	Forward	5′-TCCCAGCTACTCGGTAGCCT-3′
	Reverse	5′-GTGAATGAAAGCACCCTGGA-3′
7	Forward	5′-TCTCAGAATTACTTGGAATTGAGG-3′
	Reverse	5′-GCCTTTTATTTTTTGCATTAAACA-3′
8–9	Forward	5′-TGGCACAAGAAAATGTGGTAACAA-3′
	Reverse	5′-TGCAGTAGAAGGGAAGAGACTAAAGA-3′
10	Forward	5′-CTCATTTTTTAGTGGGGTCAAAGG-3′
	Reverse	5′-CCTAAGCCAGCTCTTCTTCCCT-3′
11	Forward	5′-GCCTGGAACACAGTAAGCATGG-3′
	Reverse	5′-GTTCCATCGGCTGTGTACAGTG-3′
12	Forward	5′-CTATGACTGGCCGACTGGAACA-3′
	Reverse	5′-GGTCTCCTCTTGGAGGACAGAGAT-3′
13	Forward	5′-TGCTCTCTTTGTTTAACGAGTGAC-3′
	Reverse	5′-ACAACTTTTTCCTCTGTGCTTGTA-3′
14	Forward	5′-ACTATGTTGGCCAGGCTGGT-3′
	Reverse	5′-TTGTTGGCCAGGCTGATCTT-3′
15	Forward	5′-AGGACGATCACTTGAGACGAAGAG-3′
	Reverse	5′-TCCCAACTCCTGTAGGAAGAAATC-3′
16	Forward	5′-AAGGCTCTTCCTGCGTAAGACTGT-3′
	Reverse	5′-GGGTGGTCACTAATTTCAGCACAG-3′

### Cell Experiment

#### Cell Lines and Cell Culture

The human MCL cell line of Jeko-1 was provided by Shanghai Institute of Materia Medica, Chinese Academy of Sciences (Shanghai, China). The cells were cultured in RPMI-1640 medium with 20% fetal bovine serum in an incubator containing 5% CO2 at 37°C. The medium was refreshed every 3 days.

#### Plasmids

Plasmids pLKO.1-puro-SAMHD1-*sh*RNA1 and 2 were constructed for the silencing of SAMHD1. The sequences of *sh*RNA were as follows: *sh*RNA1: CCGGGGAGAGGAAGAAGCTGCTTAGCTCGAGCTAAGCAGCTTCTTCCTCTCCTTTTTG (forward) and AATTCAAAAAGGAGAGGAAGAAGCTGCTTAGCTCGAGCTAAGCAG CTTCTTCCTCTCC (reverse); *sh*RNA2: CCGGGCTGGGAGGTGGTTACTATGTCTCGAGACATAGTAACCACCTCCCAGCTTTTTG (forward) and AATTCAAAAAGCTGGGAGGTGGTTACTATGTCTCGAGA CATAGTAA CCACCTCCCAGC (reverse).

#### Western Blot

Cells were lysed with RIPA buffer (Key GEN Biotech, Nanjing, China) and the protein concentration was quantified by the bicinchoninic acid method. An equal amount of proteins was loaded and separated by 4%–10% SDS-PAGE at 180 V for 30 min and then transferred to polyvinylidene difluoride (PVDF) membranes. After being blocked with 5% BSA (bovine serum albumin) for 1 h, the membranes were incubated with primary antibodies overnight at 4°C and then with secondary antibodies (1:5,000, goat anti-rabbit/mouse IgG-HRP, Wanleibio, Shanghai, China) for 1 h at room temperature. The membranes were washed four times with TBST (Tris-buffered saline, 0.1% Tween 20) after each step described above. Specific protein bands were visualized by enzyme chemiluminescence assay. The primary antibodies used included mouse anti-SAMHD1 (Abcam, Cambridge, MA, USA), rabbit anti-caspase3/cleaved-caspase3, rabbit anti-Ki-67, and rabbit anti-β-actin (Wanleibio, Shanghai, China).

#### Cell Apoptosis Analysis

Wild-type Jeko-1 and *sh*RNA-mediated SAMHD1-silenced Jeko-1 cells infected with *sh*SAMHD1-1 or *sh*SAMHD1-2 vectors were seeded into 12-well plates at a density of 1 × 10^5^/ml for 72 h and then washed twice with PBS. Each sample was resuspended with 100 µl buffer and mixed with 5 µl Annexin V and 5 µl propidium iodide (BD Biosciences, San Jose, CA, USA). Samples were incubated in the dark at room temperature for 15 min and then supplemented with 200 µl PBS before flow cytometry analysis.

#### Cytarabine IC50

Eighty microliters of wild-type and SAMHD1-silenced Jeko-1 cell suspensions at a density of 1 × 10^5^/ml were seeded into 96-well plates, respectively. Cytarabine was five-fold serially diluted at a maximum concentration of 10 µM. Twenty microliters of cytarabine solution with different concentrations was added to the corresponding wells. DMSO and RPMI-1640 were used as positive and blank control, respectively. The cells were cultured for 72 h in an incubator at 37°C with 5% CO2. Then, each well of 96-well plates was added with 20 µl of CCK-8 and incubated for another 3 h at 37°C. The absorbance of each well at 450 nm wavelength was measured at the end of the culture.

### Statistical Analyses

OS was calculated from the date of treatment until death or the last follow-up date. PFS was counted from the date of treatment until the disease relapse or progression or the date of death from any other causes. Fisher’s exact test or the chi-square test was used to determine statistically significant difference between the clinical characteristics of the two groups. Survival was estimated using the Kaplan–Meier and evaluated by log-rank test. The effects of potential prognostic variables on survival were assessed according to the Cox regression method. *p-*value <0.05 was considered statistically significant. All calculations were performed using the IBM SPSS 22.0.

## Results

### Pathological Characteristics

**Figure 1:**
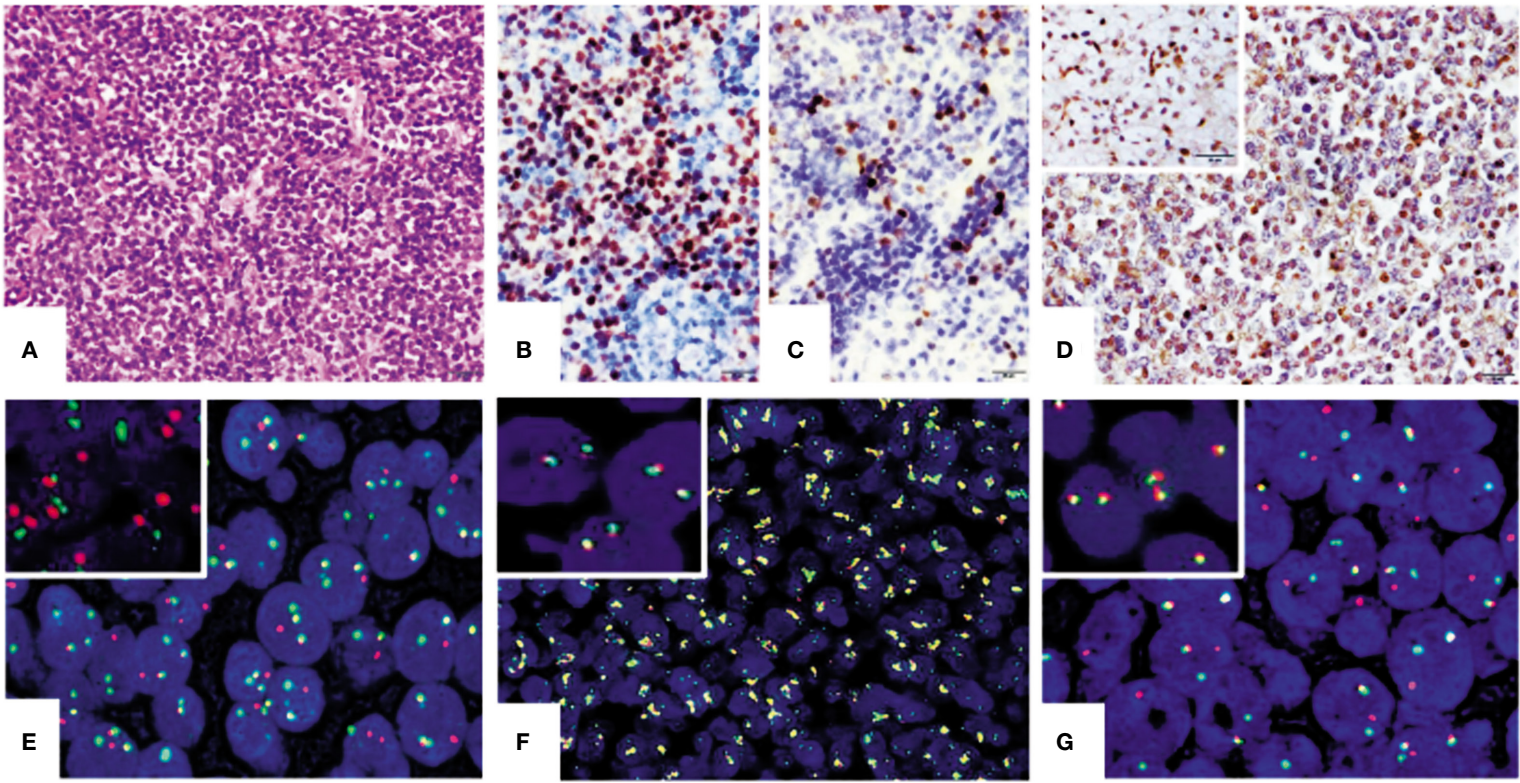
Representation of IHC and FISH results of mantle cell lymphoma (MCL) tissues. **(A)** Morphology of MCL. Tumor cells were small to medium and were diffusely arranged in the mantle and paracortex area of the lymph node (HE ×400). Panels **(B–D)** are the results of IHC staining (×400) for cyclin D1 **(B)**, SOX11 **(C)**, and SAMHD1 in MCL tissues **(D)** and the insert in **(D)** is SAMHD1-positive control from clear cell kidney carcinoma. Panels **(E–G)** (×1,000) are examples of positive FISH tests of t(11;14) IGH/CCND1 translocation and cytokine gene rearrangement in MCL tissues. Panel **(E)** shows positive for t(14;18) IGH/CCND1 translocation and panel **(G)** is positive for CCND1 gene rearrangement. CCND2 gene amplification was also detected in one case of MCL **(F)**. Inserts in panels **(E–G)** are their corresponding negative controls.

Pathological review was performed for those 58 patients enrolled in this study. Most tumors were located in the lymph nodes in the first episode. The architecture of the lymph nodes was destructed by monomorphic small- to medium-sized tumor cells (**Figure 1A**). The diagnosis of MCL was confirmed by the expression of CD20, CD5, cyclin D1 (**Figure 1B**), SOX11 (**Figure 1C**), and SAMHD1 (**Figure 1D**) of tumor cells. Cyclin D1 was positive in 55 of 58 (94.8%) patients, and CD5 was positive in 36 of 58 (62%). In confirmed MCL, SOX11 expression was tested in all cases. Three were negative for SOX11. Two of them had either blastoid morphology or p53 protein expression and both had a high Ki-67 index. Ki-67 quantification revealed 19/58 (32.8%) cases with a Ki-67 index ≥30%.

### Fusion Translocation and Gene Rearrangement in MCL

These 58 MCL cases along with 5 cases of reactive lymph tissue as negative controls were tested for t(11;14)(q13;q32) IGH/CCND1 translocation by FISH assay. Compared with negative control samples with two orange and two green FISH signals in fusion translocation FISH assay, a typical positive cell presented two yellow signals, one unpaired red signal, and one unpaired green signal. In FISH gene rearrangement assays, a negative cell showed two yellow signals. A cell with a typical positive FISH signal pattern for CCND2 or 3 gene rearrangement had one yellow, one red, and one green signal. The yellow signals could be created by two overlaid or very closely positioned orange and green signals or fusion signals. The cutoff level was 11.5% for the translocation FISH assay and 5.3% for the gene rearrangement assay. Results showed that 49 out of 58 MCL cases (84.5%) were positive for t(11;14)(q13;q32) IGH/CCND1 translocation (**Figure 1E**). The remaining nine negative cases were further tested for CCND2 and CCND3 gene rearrangement. Four of them were positive for CCND2 gene rearrangement and three were positive for CCND3 gene rearrangement (**Figure 1G**). Two cases showed the amplification of the *CCND2* gene (**Figure 1F**).

### *SAMHD1* Expression and Gene Mutations in MCL

All enrolled MCL cases were tested for the SAMHD1 expression by IHC. Results showed that SAMHD1 was positive in 36/58 (62.1%) patients, with strong positive in 12 (20.7%) patients, moderate positive in 8 (13.8%) patients, and weak positive in 16 (27.6%) patients (**Figures 1A–D**).

**Figure 2:**
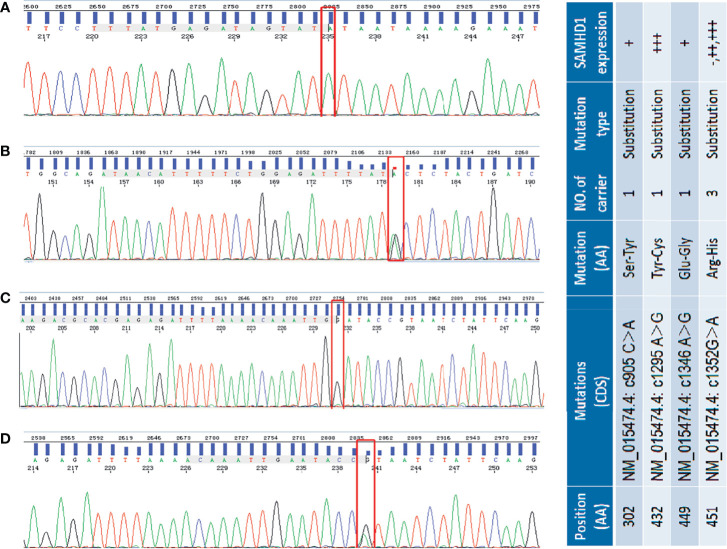
Selected sequencing chromatograms of *SAMHD1* using Sanger sequencing. Panel **(A)** shows the homogeneous c905 C>A mutation (red box highlighted) in exon 8 of *SAMHD1*. Panel **(B)** shows the heterogeneous c1295A>G transition in exon 12 of *SAMHD1*. In panel **(C)**, the highlighted red box represents the homogeneous c1346G>A transition in exon 12 of *SAMHD1*. Panel **(D)** is the heterogeneous c1552G>A mutation in exon 12 of *SAMHD1.* The included table on the right is the correlation of SAM HD1 mutations and their expression in MCL cases.

The *SAMHD1* gene is located on chromosome 20q11.23 and contains 16 exons. The results of Sanger sequencing of 58 MCL samples revealed that *SAMHD1* mutations were mainly distributed in the regions of exons 8 and 12. Four missense mutations (NM_015474.4: c905C>A, c1295A>G, c1346A>G, and c1352G>A) (**Figures 2A–D**) were found in five patients. These four missense mutations have not been described previously and are associated with amino acid alterations. We have submitted these mutation sequences to GenBank. Their accession numbers are MW893458 (c905C>A), MW893459 (c1346A>G), and MW893460 (c1295A>G and c1352G>A). Although the mutation c1346A>G has not been described in human, it has been reported in *Nomascus leucogenys* (NCBI Reference Sequence: NM_001280119.1) (36).

Three of the five patients shared the same c1352G>A mutation which leads to the amino acid change from arginine to histidine (R451H). However, these three patients presented different SAMHD1 protein expression profiles in the IHC tests. One patient showed no SAMHD1 expression. Another case had a moderate increase of SAMHD1 expression. The other one who had an additional c1295A>G mutation associated with tyrosine to cysteine (Y432C) protein sequence change, besides the R451H alteration, showed strong SAMHD1 positivity. In addition, two single nucleotide mutations, c905C>A and c1346A>G associated with amino acid change from serine to tyrosine (S302Y) and glutamate to glycine (E449G), respectively, were found in two different patients with a low SAMHD1 expression. The association between the present genetic mutations of SAMHD1 and the expression in MCL protein was not significant.

### *SAMHD1* Correlated With MCL Cell Proliferation, Apoptosis, and Sensitivity to Cytarabine

**Figure 3:**
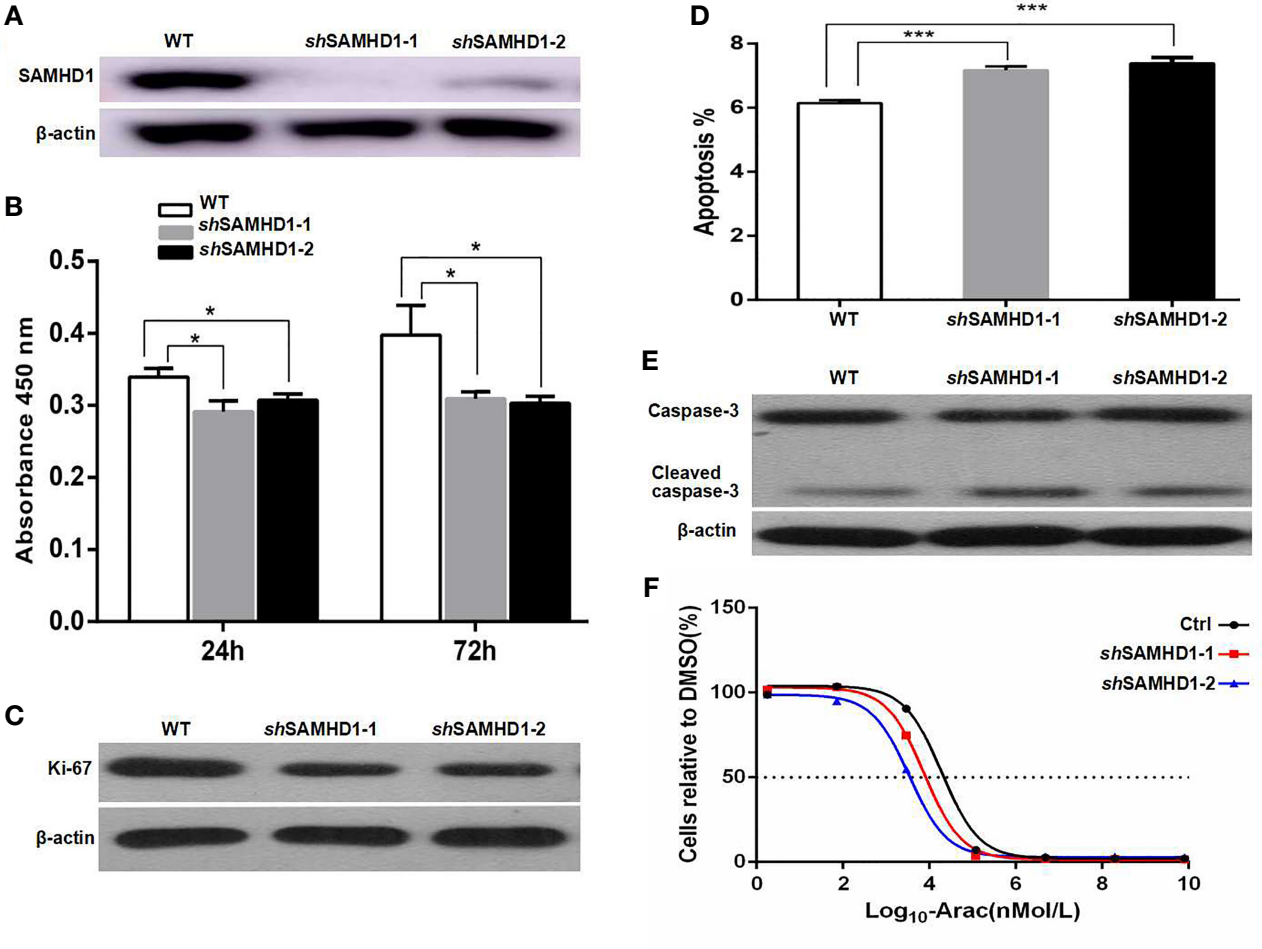
SAMHD1 promotes the proliferation, inhibits the apoptosis, and mediates the resistance to cytarabine of MCL cells. MCL cells (Jeko-1) were cultured and their *SAMHD1* gene was silenced by using two lentiviral vectors SAMHD1-*sh*RNA1 and 2 to generate two SAMHD1-silenced Jeko-1 cell lines (*sh*SAMHD1-1 and *sh*SAMHD1-2). The expression of SAMHD1 in wild-type Jeko-1 cells and two *SAMHD1*-silenced Jeko-1 cell lines detected by Western blot **(A)**. The proliferation of Jeko-1 cells. Wild-type Jeko-1 cells showed higher proliferation activity than two *SAMHD1*-silenced cell lines, *sh*SAMHD1-1 and *sh*SAMHD1-2 **(B)**. Panel **(C)** is the Western blot of Ki-67 expression. Wild-type Jeko-1 cells expressed a higher level of Ki-67 protein than the two silenced cell lines, *sh*SAMHD1-1 and *sh*SAMHD1-2. *SAMHD1*-silenced Jeko-1 cells (both *sh*SAMHD1-1 and *sh*SAMHD1-2) had a higher proportion of apoptosis cells than the wild-type parent cell **(D)**. **(E)** The expression of caspase-3 in the SAMHD1-1 silenced cells was the same as in wild-type Jeko-1 cells, while the level of cleaved-caspase-3 in the silenced cells was higher than the wild-type cells **(E)**. IC50 of cytarabine for *sh*SAMHD1-1 and *sh*SAMHD1-2 cells was lower than that for wild-type Jeko-1 **(F)**, indicating that SAMHD1 promoted cytarabine resistance. The symbol "*" represents p < 0.5, and "***" represents p < 0.001.

The MCL cell line Jeko-1 was used to investigate the role of SAMHD1 in cell proliferation, apoptosis, and resistance to cytarabine of MCL. SAMHD1 in Jeko-1 cells was silenced using two lentiviral vectors that encoded SAMHD1-specific *sh*RNA named as *sh*SAMHD1-1 and *sh*SAMHD1-2, respectively (**Figure 3A**). The cell proliferation was decreased in SAMHD1-silenced Jeko-1 compared with its mock-transfected control wild-type (WT) cells (**Figure 3B**). On the other hand, Ki-67 expression was lower in *sh*SAMHD1-1 and SAMHD1-2 cells than in the WT Jeko-1 cells (**Figure 3C**). The Jeko-1 cells with *sh*SAMHD1-1 and *sh*SAMHD1-2 had high apoptotic rate and increased expression of cleaved-caspase-3. These results suggest that SAMHD1 has anti-apoptotic effect (**Figures 3D, E**). The half maximal inhibitory concentration (IC50) of cytarabine for Jeko-1 cells was decreased after the cells were infected with *sh*SAMHD1-1 and *sh*SAMHD1-2 vectors, and the SAMHD1 expression decreased. These results indicate that the sensitivity of Jeko-1 cells to cytarabine was increased when SAMHD1 was silenced (**Figure 3F**). SAMHD1 may be associated with increased cytarabine resistance in MCL chemotherapy.

### *SAMHD1* With Response to Chemotherapy and Outcome

Among 58 MCL patients, there were 33 patients who received treatment in our hospital. The characteristics of these 33 patients are displayed in **Table 2**. The median age of the patients was 54 (range, 35–72) years old and 60 (range, 42–83) years old in the SAMHD1-negative and SAMHD1-positive groups, respectively (*p* = 0.275). Male patients accounted for 69% in the SAMHD1-negative group and 60% in the positive group. Patients in both groups had similar LDH level, B symptoms, bone marrow involvement, ECOG, and MIPI. There were 85% patients in the SAMHD1-positive group with elevated β2-microglobulin level, compared with 54% patients in the SAMHD1-negative group (*p* = 0.009). The SAMHD1-positive group showed a lower response rate (45%) to chemotherapy than 85% in the SAMHD1-negative group (*p* = 0.023).

**Table 2:** Clinical characteristics and SAMHD1 expression in 33 cases of MCL with chemotherapy^a^.

Characteristic/outcomes	SAMHD1 (−)(*N* = 13)	SAMHD1 (+)(*N* = 20)	*p*-value
Age (years)			
<60	9 (69.23%)	10 (50.00%)	
≥60	4 (30.77%)	10 (50.00%)	0.275
Gender			
Male	9 (69.23%)	12 (60.00%)	
Female	4 (30.77%)	8 (40.00%)	0.719
β2-MG level			
Normal	6 (46.15%)	3 (15.00%)	
High	7 (53.85%)	17 (85.00%)	0.009
LDH level			
Normal	12 (92.30%)	18 (90.00%)	
High	1 (7.70%)	2 (10.00%)	1.000
B symptoms			
Yes	4 (30.77%)	10 (50.00%)	
No	9 (69.23%)	10 (50.00%)	0.275
BM involvement			
Yes	8 (61.54%)	13 (65.00%)	1.000
No	5 (38.46%)	7 (35.00%)	
ECOG score			
<2	6 (46.15%)	11 (55.00%)	
≥2	7 (53.85%)	9 (45.00%)	0.619
MIPI score			
Low/middle risk (0–5)	10 (76.92%)	11 (55.00%)	
High risk (6–11)	3 (23.08%)	9 (45.00%)	0.278
Chemotherapy regime			
Ara-C-containing	5 (38.46%)	6 (30.00%)	
Without Ara-C	8 (61.54%)	14 (70.00%)	0.714
Response rate (CR + PR), %	11 (85%)	9 (45%)	0.023*

LDH, lactate dehydrogenase; β2-MG, β2-microglobulin; BM, bone marrow; ECOG, Eastern Cooperative Oncology Group; MIPI, mantle cell lymphoma International Prognostic Index; Ara-C, cytarabine; OS, overall survival; PFS, progression-free survival; CR + PR, complete remission and partial remission.

a In accordance with the World Health Organization classification (33). *p < 0.05.

**Figure 4:**
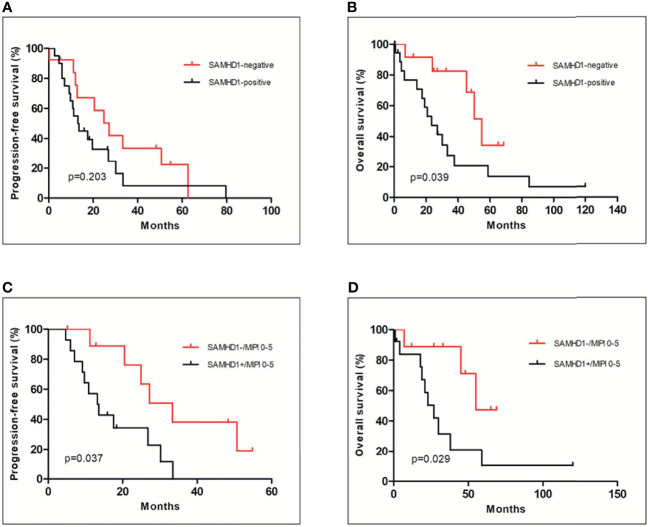
Relationship of the SAMHD1 expression with clinical outcome of MCL patients. There was no significance in PFS between SAMHD1-positive and SAMHD1-negative patients **(A)**. However, SAMHD1-positive patients had shorter OS than SAMHD1-negative patients **(B)**. Among low- to intermediate-risk MCL patients (MIPI score between 0 and 5), SAMHD1-positive patients had worse PFS **(C)** and OS **(D)** than SAMHD1-negative patients.

The univariate analysis was performed based on SAMHD1 expression, Ki-67, or MIPI. The median PFS of SAMHD1-positive and SAMHD1-negative groups was 12 (range, 2–80) months and 26 (range, 11–61) months (*p* = 0.203), respectively (**Figure 4A**). The OS of SAMHD1-negative patients was 48 (range, 12–87) months and was much longer than 18 (range, 2–119) months (*p* = 0.039) in SAMHD1-positive patients (**Figure 4B**). The patients with SAMHD1 expression had a significantly worse clinical outcome. Patients with a higher Ki-67 proliferative index (*p* = 0.007) and MIPI score (*p* = 0.0322) also predicted poor prognosis. If the SAMHD1 expression was combined with MIPI score, the PFS (*p* = 0.037) and OS (*p* = 0.029) of MCL patients with SAMHD1 positive and MIPI score more than 5 were much lower and the outcome of the patients was poorer compared with SAMHD1 negative with MIPI score less than 5 (**Figures 4C, D**).

## Discussion

The treatment algorithm for MCL has been changed significantly over the past three decades. Several treatment approaches for MCL have been developed (15–20). RDHAP (rituximab, dexamethasone, high-dose cytarabine, platinum) regimen or alternating RCHOP and RDHAP is suggested for induction therapy, and high-dose chemotherapy followed by autologous stem cell transplantation is recommended for consolidation therapy (21, 25). As part of alternative treatment regimens for MCL, cytarabine has shown its effectiveness against relapsed or refractory MCL (23, 24). The possibility of achieving equal long-term clinical outcomes of cytarabine-based regimens has become a key clinical question in the initial management of MCL.

SAMHD1 is responsible for maintaining cellular dNTP balance during cell cycle and regulating cell proliferation (5). It was identified as a driver gene of MCL development (37). SAMHD1 was reported as a barrier to cytarabine and a biomarker of grim prognosis for AML patients (12). SAMHD1 could hydrolyze Ara-CTP in leukemic cells, resulting in the reduction of cellular Ara-CTP concentration and attenuation of the efficacy of the drug (13, 14). Thus, high SAMHD1 expression might compromise chemotherapeutic agent potency (38). Cytarabine is commonly used to treat MCL in standard chemotherapy regimens (21, 22). However, the SAMHD1 status in MCL has not been well established previously. In a clinical setting, most hematologists make their clinical treatment decisions for MCL based on MIPI that does not include SAMHD1 status. This study assessed the SAMHD1 expression and gene structure (mutations) in MCL tissues and analyzed the association between SAMHD1 expression and chemotherapy response as well as the clinical outcome of patients. Fifty-eight MCL samples were used in this study for the SAMHD1 expression by IHC and gene mutation analysis by Sanger sequencing. Results showed that the status of SAMHD1 expression was different among the MCL patients. In 33 MCL patients with chemotherapy, the treatment with cytarabine did not show a significant impact on the PFS of patients. The reason may be attributed to limited patients enrolled. Further study with more patients and a stricter design is needed on this subject.

Among low- to intermediate-risk MCL patients (MIPI score between 0 and 5), our study revealed that SAMHD1-positive patients had worse PFS and OS compared with SAMHD1-negative patients. Importantly, the SAMHD1-postive group showed more chemotherapy resistance and shorter OS in comparison with the SAMHD1-negative group.

At the same time, the present study has found four new missense *SAMHD1* mutations associated with amino acid alteration in five patients. Among these five cases, three patients shared the same A82G mutation which leads to the change of an amino acid from valine to isoleucine (Val-Ile). Unfortunately, these three patients presented different SAMHD1 expression profiles. In addition, two single nucleotide mutations of C53A and A76G associated with amino acid change from serine to tyrosine (Ser-Tyr) and asparagine to aspartic acid (Asn-Asp), respectively, were found in two different patients with a low SAMHD1 expression. In the publicly available database of the Cancer Genome Atlas (TCGA), 444 *SAMHD1* somatic mutations were found in cancers varying from hematological malignancy to solid tumors. Four mutations were reported in AML and DLBCL. The present four gene mutations of *SAMHD1* in MCL discovered in this study have not been listed in the TCGA database and have not been found in available literature. Therefore, this is the first time that these four missense mutations of *SAMHD1* were found in human malignancies. However, one (c1346A>G) of the four mutations has been reported in *N. leucogenys*. The significance of sharing the same sequence at this position between human and *N. leucogenys* is unknown. In addition, we did not find a significant correlation between mutations and *SAMHD1* expression in MCL and between mutations and clinical outcome. Although the newly found mutations had no obvious effect on the prognosis of MCL patients, some *SAMHD1* mutations previously reported were associated with chronic lymphocytic (B-cell) leukemia development (11) and changed its dNTPase activity (39). It will be very interesting to study the impact of these four new mutations we found in this study on the structure and function of the SAMHD1 molecule. Further study is also desired to clarify the role of these *SAMHD1* mutations in MCL development and progression. Besides mutations, it has been reported that *SAMHD1* promoter methylation could also upregulate SAMHD1 expression and *SAMHD1* acetylation can enhance dNTP hydrolytic activity (40, 41). Therefore, the promoter and not the mutations of a gene, unless there are some nonsense mutations, determines the level of gene expression. It could be the reason why the mutations we found in this study were not correlated with the expression level of the *SAMHD1* gene.

To investigate the role of SAMHD1 in cell proliferation, apoptosis, and resistance to cytarabine of MCL cells, the *SAMHD1* gene was silenced in the MCL cell line Jeko-1 using two lentiviral vectors (*sh*SAMHD1-1 and *sh*SAMHD1-2). The cell proliferative rate and Ki-67 expression were decreased in Jeko-1 transfected with *sh*SAMHD1-1 or *sh*SAMHD1-2 compared with mock-transfected control cells. Jeko-1 cells with *sh*SAMHD1-1 and *sh*SAMHD1-2 had a higher rate of apoptosis and cleaved-caspase-3 level than the control cells, which implied that SAMHD1 had anti-apoptotic effect. Specially, Jeko-1 with *sh*SAMHD1-1 and *sh*SAMHD1-2 showed lower IC50 to cytarabine, indicating that the MCL cell line Jeko-1 had higher sensitivity to cytarabine after the *SAMHD1* gene was silenced. These results indicate that SAMHD1 inhibits MCL cell proliferation and apoptosis and might be associated with cytarabine resistance of MCL to chemotherapy.

Additionally, we analyzed the correlation between the cyclin D1 expression; rearrangement of cyclin D1, D2, and D3; and SAMHD1 expression or mutations, and the results showed that all the SAMHD1 strong expression or mutation cases have high cyclin D1 expression and rearrangement of the gene. MCL diagnosis is based on the expression and/or rearrangement of cyclin D1. However, we did not find a significant correlation of the cyclin D1 expression and rearrangement with SAMHD1 expression and mutation, partially due to limited MCL cases with rearrangement of cyclin D2 and D3 for the analysis. However, there was an interesting relationship between CD5 and SAMHD1 expression. The cases of SAMHD1 strong expression were also CD5 positive, while SAMHD1-negative cases had low or negative CD5 expression. Though CD5 is a useful biomarker of MCL, only some MCL cases are CD5 positive. Therefore, we recommend using both SAMHD1 and CD5 to assess the MCL outcome in clinical practice.

In conclusion, our results revealed that the majority of MCLs were characterized by a high expression of SAMHD1 and a poor response to chemotherapy. The SAMHD1 level was correlated to MCL cell proliferation and apoptosis and associated with cytarabine resistance during chemotherapy. The silencing of the *SAMHD1* gene leads to increased apoptosis, decreased proliferation, and cytarabine resistance of the cancer cells. This study also discovered four new *SAMHD1* mutations in MCL based on the databases of TCGA and COSMIC. However, we did not get the correlation between these mutations and SAMHD1 expression or the clinical outcome of MCL patients due to limited SAMHD1 cases. Together with MIPI and Ki-67 results, SAMHD1 should be considered an important marker for MCL patient risk stratification in clinical management. Furthermore, we recommend that SAMHD1 and CD5 should be used together for the assessment of MCL patient outcome.

## Data Availability Statement

The datasets presented in this study can be found in online repositories. The names of the repository/repositories and accession number(s) can be found below: GenBank and MW893458, MW893459, and MW893460.

## Ethics Statement

The studies involving human participants were reviewed and approved by Changhai Hospital Ethics Committee. The patients/participants provided their written informed consent to participate in this study.

## Author Contributions

MH designed the study, analyzed the data, and revised the manuscript. TW provided statistical analysis and wrote the draft of the manuscript. LJ performed the molecular testing. JMY supervised the study and reviewed the manuscript. WY, XL, SY, JC, and LG helped in analyzing the results and giving suggestions. All authors contributed to the article and approved the submitted version.

## Funding

This work was supported by the National Natural Science Foundation of China (NSFC 81770209) and National Science and Technology Major Project of China (No. 2017ZX09304-021).

## Conflict of Interest

The authors declare that the research was conducted in the absence of any commercial or financial relationships that could be construed as a potential conflict of interest.

## Publisher’s Note

All claims expressed in this article are solely those of the authors and do not necessarily represent those of their affiliated organizations, or those of the publisher, the editors and the reviewers. Any product that may be evaluated in this article, or claim that may be made by its manufacturer, is not guaranteed or endorsed by the publisher.
